# Effect of Interindividual Variability in Metabolic Clearance and Relative Bioavailability on Rifampicin Exposure in Tuberculosis Patients with and without HIV Co-Infection: Does Formulation Quality Matter?

**DOI:** 10.3390/pharmaceutics16080970

**Published:** 2024-07-23

**Authors:** Glauco Henrique Balthazar Nardotto, Elin M. Svenson, Valdes Roberto Bollela, Adriana Rocha, Svetoslav Nanev Slavov, João Paulo Bianchi Ximenez, Oscar Della Pasqua, Vera Lucia Lanchote

**Affiliations:** 1 Faculdade de Ciências Farmacêuticas de Ribeirão Preto, Universidade de São Paulo, Ribeirão Preto 14040-903, Brazil; glauconardotto@gmail.com (G.H.B.N.); drirocha@fcfrp.usp.br (A.R.); joaopaulo.ximenez@usp.br (J.P.B.X.); 2 Department of Pharmacology and Therapeutics, Roswell Park Comprehensive Cancer Center, Buffalo, NY 14263, USA; 3 Department of Pharmacy, Uppsala University, 75123 Uppsala, Sweden; elin.svensson@farmaci.uu.se; 4 Faculdade de Medicina de Ribeirão Preto, Universidade de São Paulo, Ribeirão Preto 14049-900, Brazil; vbollela@fmrp.usp.br; 5 Center for Viral Surveillance and Serological Evaluation-CeVIVAs, Butantan Institute, Sao Paulo 05503-900, Brazil; svetoslav.slavov@fundacaobutantan.org.br; 6 Clinical Pharmacology & Therapeutics Group, University College London, London WC1J 9JP, UK; o.dellapasqua@ucl.ac.uk

**Keywords:** pulmonary tuberculosis, human immunodeficiency virus, rifampicin, 25-O-desacetyl-rifampicin, population pharmacokinetics, bioavailability

## Abstract

The present study aims to characterise the pharmacokinetics of rifampicin (RIF) in tuberculosis (TB) patients with and without HIV co-infection, considering the formation of 25-O-desacetyl-rifampicin (desRIF). It is hypothesised that the metabolite formation, HIV co-infection and drug formulation may further explain the interindividual variation in the exposure to RIF. Pharmacokinetic, clinical, and demographic data from TB patients with (TB-HIV+ group; *n* = 18) or without HIV (TB-HIV− group; *n* = 15) who were receiving RIF as part of a four-drug fixed-dose combination (FDC) regimen (RIF, isoniazid, pyrazinamide, and ethambutol) were analysed, along with the published literature data on the relative bioavailability of different formulations. A population pharmacokinetic model, including the formation of desRIF, was developed and compared to a model based solely on the parent drug. HIV co-infection does not alter the plasma exposure to RIF and the desRIF formation does not contribute to the observed variability in the RIF disposition. The relative bioavailability and RIF plasma exposure were significantly lower than previously reported for the standard regimen with FDC tablets. Furthermore, participants weighting less than 50 kg do not reach the same RIF plasma exposure as compared to those weighting >50 kg. In conclusion, as no covariate was identified other than body weight on CL/F and Vd/F, low systemic exposure to RIF is likely to be caused by the low bioavailability of the formulation.

## 1. Introduction

Tuberculosis (TB) remains the world’s second leading cause of death from a single infectious agent after COVID-19, and it causes almost twice as many deaths when compared to HIV [1]. However, the immunodeficiency associated with HIV appears to contribute to co-infection, which results in a significant proportion of HIV-positive subjects developing active TB. In addition, epidemiological data also show that treatment failure and poorer outcomes are higher in HIV-positive subjects [2,3,4].

Even though such findings must be considered within a much broader context, limited attention has been paid to the variability in drug exposure in subjects who are being treated with antiretrovirals and antitubercular drugs, which are known to have no or minor metabolic interaction. Consequently, an important question to be addressed is the correlation between TB treatment failure and pharmacokinetic variability in subjects with HIV/TB co-infection. Rifampicin (RIF) is an essential component of the first-line anti-tuberculosis drug therapy. Given the significant effect of body weight on RIF disposition, the World Health Organization recommends the use of weight-banded dosing [5,6]. RIF is usually administered daily as part of a four-drug fixed-dose combination (FDC) regimen for TB treatment (RIF, isoniazid, pyrazinamide, and ethambutol). Whilst weight-banded dosing facilitates interventions and an FDC reduces the pill burden, improving adherence to treatment, high intra- and interindividual variability in RIF exposure is observed following therapeutically recommended doses [7,8,9,10,11,12]. There are many reports on the interindividual variability (IIV) of RIF pharmacokinetics, which attribute it to the formulation type [13], age [14], sex [13,15], HIV co-infection [16], weight (or other body size descriptor) [14,17,18,19,20], and comedications [17]. Despite adequate information on the summary of product characteristics regarding the effect of such factors, interindividual variability in the systemic exposure to RIF remains high [16,17,18,19,20,21,22,23,24,25,26,27], leading to a growing consensus that higher RIF doses are required to ensure efficacy [8,10,12]. However, there is disagreement on whether HIV co-infection, i.e., the potential effect of HIV-related inflammation and changes in the immune response, affects the pharmacokinetics of RIF, and consequently, whether dose adjustment should be considered for TB patients living with HIV [7,16,28]. 

According to the Biopharmaceutical Classification System, RIF is a class II drug presenting low water solubility and high permeability [29]. Its variable bioavailability is mainly related to the formulation dissolution and disintegration properties [15]. In addition, considering that HIV affects mucosal surfaces with inflammation independently of the viral load [30], it is conceivable that RIF’s absorption characteristics related to both the rate and extent of the absorption may be affected in subjects with HIV/TB co-infection.

RIF is eliminated primarily by hepatic mechanisms, but 13–24% of unchanged drug is eliminated renally [31,32]. RIF is metabolised via β-esterase or other esterases in liver microsomes [31,33] to 25-desacetylrifampicin (desRIF, a major contributor) and excreted in bile. At therapeutic doses, RIF’s pharmacokinetics is nonlinear (10–40 mg/kg daily), probably due to the saturable active secretion into the bile. However, the transporter involved in this process is unknown [20]. In addition, autoinduction of enzymes and/or transporters leads to a significant decrease in RIF exposure over time. Previous studies have shown that 90% of the maximum induction is reached after two weeks of RIF daily treatment [34]. Thus, RIF pharmacokinetics shows concentration and time-dependent elimination and dose-dependent bioavailability [20]. Moreover, possibly, RIF is a substrate of the efflux transporter P-glycoprotein (P-gp), encoded by the ABCB1 gene [35], and of the organic anion-transporting polypeptide 1B1 (OATP1B1), encoded by the *SLCO1B1* gene [36,37].

Considering that desRIF shows between 50 and 100% of the antimicrobial activity of RIF against *Mycobacterium tuberculosis* [34], it would be of interest to understand whether differences in the metabolite formation contribute to the overall variability in the systemic exposure to RIF, and consequently, whether different metabolic phenotypes may be associated with poorer outcome in patients with HIV/TB co-infection.

Here, we attempt to quantify the effect of interindividual differences in the desRIF formation and HIV-co-infection on the variability in exposure to RIF, taking into consideration the potential contribution of formulation-related differences in the relative bioavailability. A model-based approach is proposed, in which the parent drug and metabolite are evaluated together and separately [34]. Previously, two population pharmacokinetic studies of RIF were developed with the inclusion of desRIF, one in healthy Asian subjects following rifampicin (600 mg) daily treatment for 14 days [17] and another in patients co-infected with TB and HIV [38]. However, these authors have not considered the potential confounding factor of geographical ancestry and the analysis has been limited to Asian and African, or Latin American, populations [13,39,40]. 

The present study aims to evaluate the pharmacokinetics of RIF in TB patients from Southeast Brazil with and without HIV co-infection, considering the formation of the metabolite desRIF. It is hypothesised that the metabolite formation, HIV co-infection and drug formulation may further explain the interindividual variation in RIF plasma exposure. 

## 2. Materials and Methods

### 2.1. Clinical Study

The study protocol was approved by the local Hospital Research Ethics Committee (CEP/FCFRP n°: 405, Process number: 032398/2016), and all the patients signed the informed consent form. This investigation was conducted in accordance with the Declaration of Helsinki and national and institutional standards.

HIV-negative and HIV-positive subjects who were diagnosed with TB (TB-HIV− group, *n* = 15; TB-HIV+ group, *n* = 18) were enrolled after they had started the second month of the standard of care therapy. The TB-HIV− and TB-HIV+ groups consisted of 4 female/11 male and 1 female/17 male subjects, respectively. Their age ranged between 18 to 60 years, whereas the body weight of the TB-HIV+ group (range: 38.5 to 65 kg) was lower than the TB-HIV− group (range: 43 to 85.5 kg) (*p* < 0.05; *t*-test). None of the participants were considered obese. Further details on the participants’ demographic data can be found elsewhere [41]. All the subjects were treated with FDC tablets containing rifampicin (150 mg), isoniazid (75 mg), pyrazinamide (400 mg), and ethambutol (250 mg) (Lupin LTD A-28/1, MIDC, Chikalthana, Aurangabad, India and imported by Fundação Osvaldo Cruz-Farmanguinhos, Rio de Janeiro, Brazil). The FDC tablets were administered under fasting conditions based on weight bands: 2 tablets (20–35 kg), 3 tablets (36–50 kg), or 4 tablets (>50 kg) according to the World Health Organization guidelines [5,6]. In addition, the TB-HIV+ subjects were receiving lamivudine, tenofovir (or zidovudine), and raltegravir (or efavirenz). Serial blood samples were collected over the 24 h dose interval at times zero, 0.25, 0.5, 1, 1.5, 2, 2.5, 3, 3.5, 4, 5, 6, 9, 12, 15, 18, 21, and 24 h after antibiotics administration. The plasma aliquots were stored at −80 °C until analysis and then analysed by UPLC-MS/MS as previously described [41].

### 2.2. Population Pharmacokinetic Models

The population pharmacokinetics of RIF and desRIF was evaluated by nonlinear mixed-effects modelling. To account for the effect of differences in the mass balance, the concentration data of the parent drug and its metabolite were converted into molar units. Evaluation of desRIF as a contributor to the interindividual differences in the pharmacokinetics of RIF was implemented assuming that the parent–metabolite model (RIF-desRIF) is a nested model, including the effect of body weight based on an allometrically function with fixed exponents (0.75 for CL/F and 1 for Vd/F).

Fixed and random effects were included in a stepwise manner. Parameters were estimated using the first-order conditional estimation with interaction method (FOCE-I). One- and two-compartment structural models were considered with first-order or saturable elimination, including autoinduction [18,19]. RIF absorption was modelled considering zero- or first-order absorption, whilst the lag-time was parameterised using a transit compartment [42,43]. Interindividual variability (IIV) was evaluated assuming a log-normal distribution. The residual variability was described by a proportional model with an additive error term [44,45]. As inclusion of covariates and stochastic parameters describing interindividual variability did not fully explain the observed interindividual variation in drug concentrations, random variables were used to characterise the random deviations from the variance of ε, which is assumed to be the same for all subjects. This term allowed different individuals to have residual variability of varying magnitude. Further details on the model parameterisation are included in the control stream file (Supplementary Materials).

The model-building criteria included the following: (i) successful minimisation and covariance step, (ii) acceptable values of relative standard error (RSE) and shrinkage of each estimate, (iii) number of significant digits, and (iv) acceptable gradients at the last iteration [45,46]. The comparison between hierarchical models was based on graphic and statistical methods that included (1) reduction of the objective function value (OFV) and AIC (Akaike information criteria) [47], (2) goodness of fitting plot (GOF) [45,48] and (3) visual predictive checks (VPCs) [45,48,49], posterior predictive checks (PPC) [50], normalised predictive distribution errors (NPDE) [51] and mirror plots.

The influence of continuous (age and C reactive protein) and categorical (HIV co-infection, antiretroviral treatment and *SLCO1B1* genotype) covariates other than body size on the pharmacokinetic parameters of RIF and desRIF was explored by the stepwise forward inclusion (*p* = 0.05) backward elimination (*p* = 0.01) approach according to the likelihood ratio, with that being that the difference of the -2 log-likelihood value (OFV) between two models is approximately χ^2^ distributed with degrees of freedom equal to the difference in the number of parameters between the hierarchical models [52,53]. Attention was also paid to the correlation between the stochastic parameters describing the metabolite formation rate and variability in individual RIF concentrations. Further details on the model evaluation procedures are described in the Supplementary Materials.

The maximum plasma concentration (C_max_), AUC_0–24_ (trapezoidal rule), and steady state plasma concentration (Css=AUC0−24dosing interval) were derived from the plasma concentrations over time. 

Finally, to assess the effect of formulation-related differences on systemic RIF exposure, we compared the AUC_0-24_ and bioavailability obtained with the final model with those reported previously in the literature [13,14,15,17]. This was performed by including the parameter estimates of previous RIF models as priors, as implemented in the $PRIOR NWPRI subroutine. The priors were non-informative, with the exception of RIF CL/F and V/F values, which were kept as informative priors. 

All the analyses were implemented in NONMEM v. 7.5.0 (ICON Development Solutions, Ellicott City, MD, USA) [46], using PsN v. 5.3.0 [54,55]. Data formatting and the graphical and statistical summaries were performed using R v 4.2.2 [56]. A copy of the control stream files and a summary of the model building process are presented in the Supplementary Materials.

## 3. Results

**Figure 1:**
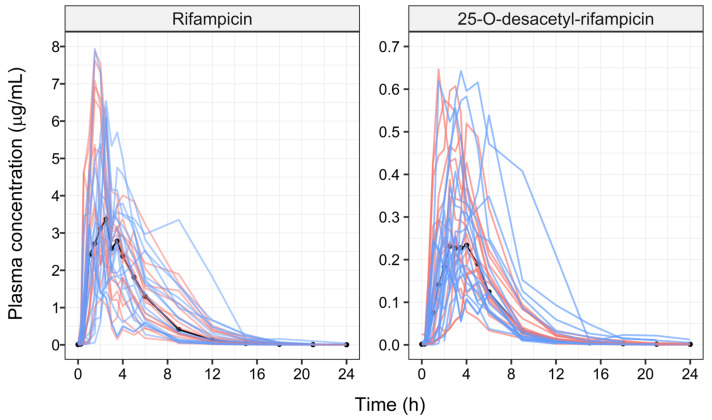
Observed rifampicin (RIF) and 25-O-desacetyl-rifampicin (desRIF) plasma concentration vs. time profiles in subjects with pulmonary tuberculosis with and without HIV co-infection (*n* = 33). The solid black line describes the median profiles. The red and blue lines depict TB-HIV− and TB-HIV+ subjects, respectively.

Figure 1 presents the observed individual concentration vs. time profiles of RIF and desRIF, as stratified by HIV co-infection. Of note was the finding that the ratio between the area under the concentration vs. time (AUC) curves between desRIF and RIF was comparable across the two groups and did not differ between individuals receiving different doses (i.e., 450 vs. 600 mg RIF).

**Figure 2:**
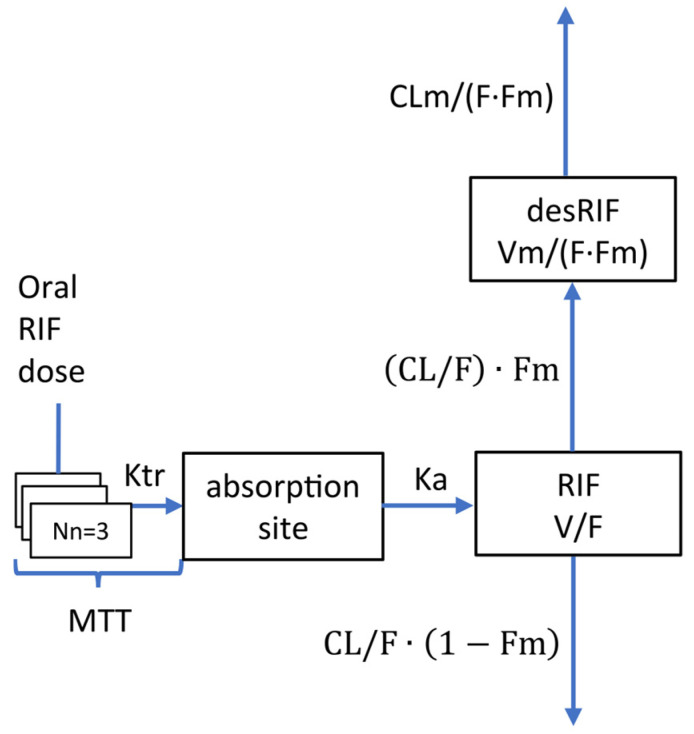
Diagram describing the structural pharmacokinetic model for rifampicin (RIF) and its metabolite 25-O-deacetyl-rifampicin (desRIF). CL/F and CLm/(F∙Fm): apparent clearance of RIF and desRIF, respectively; V/F and Vm/(F∙Fm): apparent volume of distribution of RIF and desRIF, respectively; Fm: fraction of CL/F converted into desRIF; Nn: number of transit compartments; Ka: RIF absorption rate constant; Ktr: transit absorption rate constant describing the transit time for the absorption of RIF in each compartment, MTT: mean transit time for the absorption of RIF, being: Ka = Ktr = (Nn + 1)/MTT.

The RIF and desRIF pharmacokinetics were characterized by a one-compartment model with first-order elimination. The RIF absorption was best described by the transit compartment (Nn = 3) model (Figure 2 and Table 1). The effect of body weight on the clearance and volume of distribution was implemented similarly to that outlined in previous reports, i.e., it was described by an allometric function with fixed exponents for both moieties. None of the other demographic, clinical and genotypical factors included in the covariate analysis were found to be significant.

The population estimates of the apparent clearance and volume of distribution were, respectively, 35.2 L/h and 108.0 L for RIF and 368 L/h and 226 L for desRIF (Table 1). All the parameters have been estimated with good precision; however, there was no significant reduction in the interindividual or residual variability, as indicated by the stochastic model parameters describing the pharmacokinetics of RIF. Notably, the interindividual variability in bioavailability was essential to accurately describe the individual concentration vs. time profiles of RIF and desRIF.

**Figure 3:**
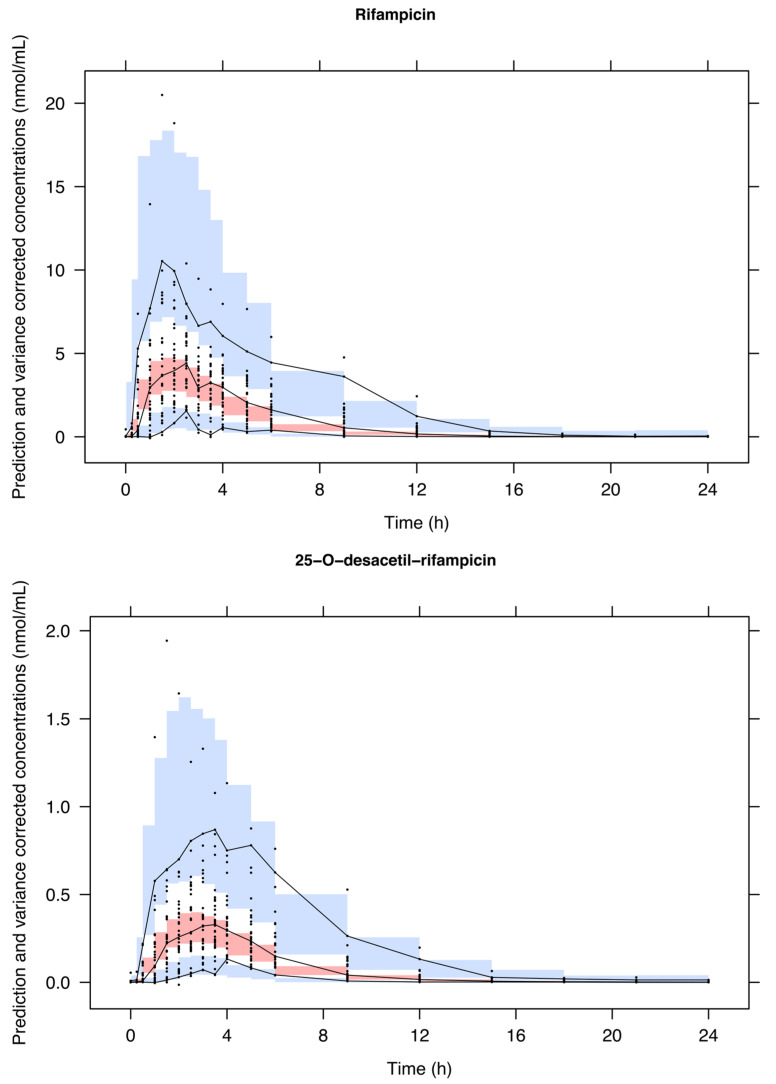
Prediction-corrected visual predictive check (VPC) of rifampicin (**upper** panel) and 25-O-desacetyl-rifampicin (**lower** panel). Dots: observed plasma concentrations. Lines: 2.5th, 50th and 97.5th percentiles of the observed concentrations vs. time profiles. Shaded areas: 2.5th, 50th and 97.5th percentiles of the simulated concentration vs. time profiles (*n* = 1000).

The VPCs of RIF and desRIF are depicted in Figure 3. As can be seen from the data scattering, the model adequately describes both moieties. Similarly, the model performance was deemed appropriate based on different diagnostic criteria, including the GOF (Figure S1). Posterior predictive checks (PPC) based on the AUC_0–24_ and C_max_ showed the accurate prediction of exposure to RIF and desRIF (Figure S2). The NPDE revealed acceptable, normally distributed errors (Figure S3). In addition, the mirror plots suggest that the variance–covariance structure was well characterised, as the simulated datasets reproduced the dispersion pattern observed in the original data (Figure S4).

Given the identification of interindividual variability in the oral bioavailability and the relatively low exposure as compared with previous studies, an assessment of the average relative bioavailability revealed marked differences across studies (Table 2 and Tables S2–S5), making it evident that the RIF concentrations achieved with the current formulation were significantly lower than those reported elsewhere [13,14,15,17].

**Figure 4:**
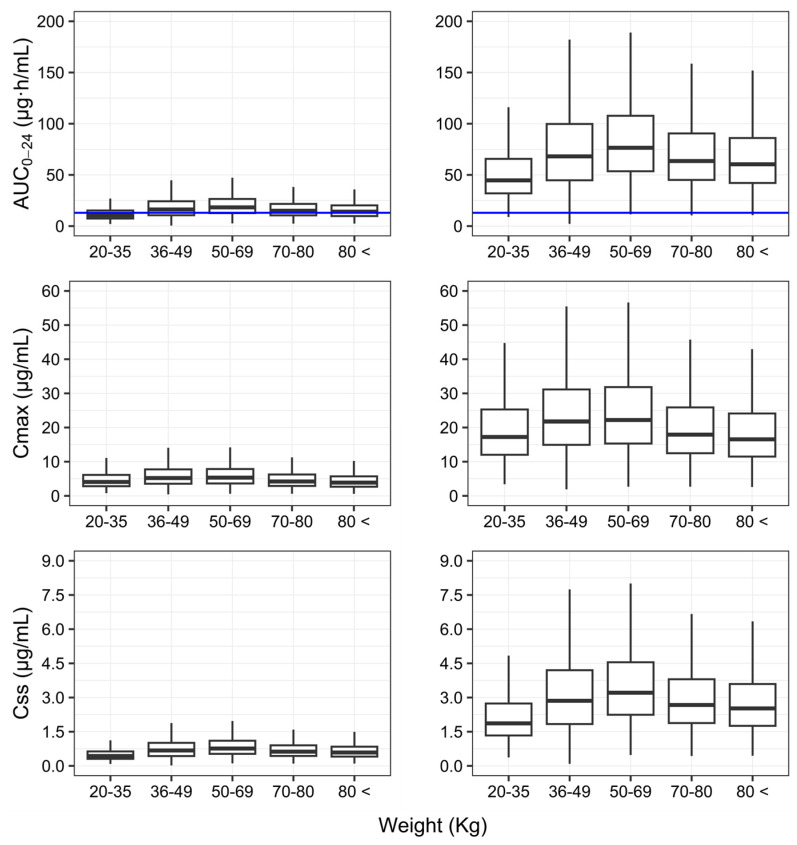
Simulated area under the plasma concentration vs. time curve 0–24 h (AUC_0–24_), maximum plasma concentration achieved (C_max_) and average steady-state concentration (Css) of rifampicin stratified by body weight (1000 replicates) using the final model (right, generic FDC formulation, Lupin LTD A-28/1, MIDC, Chikalthana, Aurangabad, India) and the one by Milán-Segovia et al., 2013 [13] (left, reference FDC product, Rifater, Sanofi-Aventis, Mexico City, Mexico). FDC: fixed-dose combination. Blue line: RIF AUC_0-24_ threshold (13 mg∙h/mL) below, which a previous report suggests to be a poor outcome [57]. FDC: fixed-dose combination. Simulations were performed using the final model and reflect the study population (*n* = 33). The weight bins were arbitrarily selected for description of the covariate effect: 20–35 kg (*n* = 5; 2 FDC tablets), 36–49 kg (*n* = 6; 3 FDC tablets), 50–69 kg (*n* = 9; 4 FDC tablets), 70–80 kg (*n* = 8; 4 FDC tablets), and >80 kg (*n* = 5; 4 FDC tablets).

Figure 4 shows how the different metrics of exposure (AUC_0–24_, C_max_, and Css) vary with body weight, and how our results compare to those of published studies including TB patients with and without HIV co-infection.

## 4. Discussion

Despite mounting evidence showing the implications of HIV/TB co-infection for treatment outcomes, there has been limited attention paid to the role of interindividual differences in exposure to anti-tubercular drugs in this group of patients. A previous report by our research group [42] showed that HIV did not influence the pharmacokinetics of RIF in TB patients. Here, we have used a model-based approach to characterise the pharmacokinetics of RIF and its active metabolite, desRIF. First, it is important to highlight that the use of a joint model including the metabolite formation and disposition did not explain the observed interindividual variability in systemic exposure to RIF. In addition, variable exposure cannot be assigned to variable adherence to treatment, as drug administration was ensured through directly observed therapy (DOT). Fasting conditions were also strictly maintained, considering that all the participants were hospitalised (inward period), excluding variability due to potential drug–food interaction. Thanks to the selected group of subjects with HIV/TB co-infection, it was possible to disentangle the potential confounding due to antiretroviral therapy from the intrinsic effect of disease (i.e., the underlying controlled viral infection, with viral load below 50 copies/mL). It is noteworthy that the TB-HIV+ patients had no gastrointestinal disorders such as diarrhoea, vomiting, opportunistic bowel infection, gastric hypoacidity, enteropathy, or comorbidities that may predispose to malabsorption [7,28,41]. None of the antiretroviral drugs showed the potential to impact systemic exposure to RIF. 

The median AUC_0–24_ desRIF/RIF ratio was approximately 0.1, a value that is very close to previously reported data [58,59]. It is also worth mentioning that given the differences in the pharmacological activity of the metabolite, the increased metabolite formation in HIV/TB co-infected subjects could have clinically relevant implications. On the other hand, it became evident that the relatively low concentration vs. plasma profiles of desRIF do not contribute appreciably to explaining the interindividual variability in RIF disposition. Even though these results may not be generalised to other metabolites, such as rifampicin glucuronide and *N*-demethyl rifampicin, it is unlikely that differences in metabolic clearance explicate the residual, random variability in the systemic exposure to RIF.

By contrast, our study reveals that the observed variation in systemic exposure between subjects is unlikely to be caused by first-pass mechanisms. Rather, it may be associated with the dosage form, with significant interindividual differences in the extent of the absorption. These differences do not seem to correlate with the baseline characteristics of the patient population, including geographical ancestry. 

Clinical studies in tuberculosis often use distinct RIF formulations from different manufactures [17,18,60,61], either as a single tablet or as an FDC [13,14,15,19,39,62]. The variation in exposure due to differences across formulations has not been evaluated, as to date no meta-analysis has been performed to assess the impact of the variation between generic formulations on systemic exposure. In addition, as bioequivalence studies are performed in healthy subjects, the interaction with other covariates has been disregarded. Unsurprisingly, disposition parameter estimates show large variation across studies and population pharmacokinetic models, including those observed in the current study. As shown in Table 1, the estimates of apparent clearance (CL/F = 35.2 L/h) and volume of distribution (V/F = 108.0 L) are significantly higher than previously reported. The CL/F was found to vary between 4.0 and 23.9 L/h, whereas the V/F between 13.8 and 77.4 L [13,14,15,20,34,41].

It is important to emphasise that a previous pharmacokinetic analysis by Schipani et al., 2016 [14], whose subjects received the same FDC tablet brand as that used in our study (Lupin Pharmaceutical Ltd., India), found relatively lower exposure and clearance estimates (CL/F = 23.9 L/h) that were already beyond the upper range reported to date (i.e., CL/F between 4.0 and 22.8 L/h) [34]. Interestingly, Milán-Segovia et al., 2013 [13] also reported that the bioavailability of RIF in Mexican subjects receiving a generic FDC tablet was only 46.8% as compared to those receiving the reference (Rifater, Sanofi-Aventis, Mexico). Previously, Milán-Segovia et al., 2010 [63] had also shown that the AUC_0−∞_ test/reference ratio of a generic FDC tablet vs. the reference formulation (Rifater, Sanofi-Aventis, Mexico) was as low as 22.08%. More recently, Medellin-Garibay et al., 2020 [39] reported that a generic FDC formulation showed bioavailability below the range required for bioequivalence (i.e., 0.85–1.25). This pattern seems to persist across different studies, which suggests that FDC formulations have lower bioavailability than RIF single tablets. It also implies that the formulation quality does not seem to be continuously monitored [15].

The issue of variable bioavailability represents a serious concern, as major efforts are being undertaken to optimise dosing regimens and reduce treatment failure in more vulnerable patients, such as those living with HIV. Yet, such an objective cannot be achieved without quality control of the standard of care medicines that are used so widely [64].

The relative bioavailability estimates (Table 2) in the current study were 32.2%, 50.1%, 70.8%, 50.3%, and 76.8% lower than that those, respectively, reported by Schipani et al., 2016 [14] (Malawian subjects taking the same FDC tablets brand as in this study; Table S2), Wilkins et al., 2008 [15] (South African subjects taking FDC tablets; Table S3), Seng et al., 2015 [17] (Asian (mainly Chinese; Table S1) subjects taking RIF + isoniazid FDC tablets), and Milán-Segovia et al., 2013 [13] (Mexican subjects taking a generic and the reference (Rifater, Sanofi-Aventis, Mexico) FDC tablets; Table S4). These differences in bioavailability have direct implications for systemic exposure, including the AUC_0–24_, C_max_ and Css (Figure 4). For instance, median estimates of the AUC_0–24_ (10.39 mg∙h/mL) in individuals with body weight <50 kg are likely to result in poor long-term outcomes, according to Pasipanodya et al. [57], who showed that RIF exposure in patients following retreatment weas ≤13 mg∙h/mL.

From a clinical pharmacology perspective, it becomes clear that any attempt to optimise regimens, so as to ensure achievement of the target exposure across the overall patient population, irrespective of differences in body weight, is pointless if the formulation quality is not warranted. For the sake of completeness, as shown in Figure 4, the effect of body weight on systemic exposure following the currently recommended weight-banded dosing regimen is minor compared to the discrepancies between formulations. 

Whilst microbiological and clinical evidence points to the importance of administering higher doses of RIF (e.g., 600 mg) to subjects who have low body weight [27], our study undoubtedly shows that a bigger issue exists, namely the consistency in bioequivalence claims supporting the commercialisation of essential medicines. In the worldwide fight against the threat of TB, and given the vulnerability of subjects living with HIV, there seems to be a gap in policy making, which offsets the advances clinical research has achieved. Accessible, cheaper medicines are crucial in the fight against TB, but medicinal products must comply with regulatory and quality standards. This requirement seems to be overlooked when considering RIF.

## 5. Conclusions

HIV co-infection does not impact plasma exposure to RIF and the desRIF formation does not contribute to the observed variability in RIF disposition. Surprisingly, our analysis allowed further investigation of the differences in the relative bioavailability, which appeared to be variable across different studies and populations, highlighting a potential quality issue for the most important component of the standard of care therapy for TB. These findings deserve further attention, as interindividual variability in exposure due to what appears to be a formulation quality issue is clinically unacceptable.

## Figures and Tables

**Table 1:** Final pharmacokinetic parameters of rifampicin (RIF) and desacetyl-rifampicin (desRIF) in subjects with pulmonary tuberculosis, with and without HIV co-infection.

	Fixed Effects	Random Effects
Parameters	Typical Value (RSE%)	CV% (RSE%)
CL/F (L/h)	35.2 (10.1)	26.9 (10.6)
V/F (L)	108.0 (9.4)	
MTT (h)	1.13 (9.0)	53.2 (7.8)
F	----	46.9 (10.7)
Nn	3 (fix)	
Fm	----	38.8 (15.8)
CLm/(F·Fm) (L/h)	368 (9.8)	
Vm/(F·Fm) (L)	226 (15.2)	63.3 (22.0)
Correlation CL/F–Fm		71.5 (2.2)
Residual variability (ε)		
RIF proportional		45.6 (8.5)
RIF additive (nmol/mL)^2^		1.85 × 10^−5^ (38.6)
η_1_ *		20.4 (37.7)
desRIF proportional		35.5 (10.1)
desRIF additive (nmol/mL)^2^		3.21 × 10^−6^ (15.7)
η_2_ *		35.1 (28.5)
$CV\%=\sqrt{exp(\omega^{2})-1}\cdot 100\;or=\sqrt{\exp\left(\varepsilon^{2}\right)-1}\cdot 100.$ * η_1_ and η_2_ are random deviations of individual i from the variance of ε, which is assumed to be the same for all subjects. RSE: relative standard error.		
Model parameterisation:		
Ka = Ktr = (Nn + 1)/MTT		
$CL/F={CL/F}_{Tipical\;Value}\cdot {\left(\frac{Weight}{55.7}\right)}^{0.75}\cdot e^{\eta }$		
$V/F={V/F}_{Tipical\;Value}\cdot \left(\frac{Weight}{55.7}\right)\cdot e^{\eta }$		
$CLm/\left(F\cdot Fm\right)={CLm/\left(F\cdot Fm\right)}_{Tipical\;Value}\cdot {\left(\frac{Weight}{55.7}\right)}^{0.75}\cdot e^{\eta }$		
$Vm/\left(F\cdot Fm\right)={Vm/\left(F\cdot Fm\right)}_{Tipical\;Value}\cdot \left(\frac{Weight}{55.7}\right)\cdot e^{\eta }$		
$F=1\cdot e^{\eta }$		
$Fm=1\cdot e^{\eta }$		

CL/F and CLm/F∙Fm: apparent clearance of RIF and desRIF, respectively; Fm: fraction of RIF that is converted into desRIF; Ka: RIF absorption rate constant; Ktr: RIF transit rate constant; MTT: mean transit time; Nn: number of transit compartments; V/F and Vm/F∙Fm: apparent volume of distribution of RIF and desRIF, respectively; WT: weight; η and ε are random variables with mean 0 and variance ω^2^ and σ^2^.

**Table 2:** Comparison of the area under plasma concentration vs. time curve from 0 to 24 h (AUC_0–24_) and the relative bioavailability of the rifampicin (RIF) formulation used in the current study and estimates obtained in previous reports (*n* = 33).

Priors Included	Frel *	Median AUC_0–24_ (25th–75th Percentiles)	AUC_0–24_ Ratio
Schipani et al., 2016 [14] FDC	0.678	30.83 (10.94–75.77)	0.620
Seng et al., 2015 [17] FDC	0.292	51.26 (16.87–143.27)	1.031
Wilkins et al. 2008 [15] FDC	0.499	28.50 (9.91–72.22)	0.573
Milán-Segovia et al., 2013 [13] Formulation A FDC	0.497	23.28 (9.42–43.11)	0.468
Milán-Segovia et al., 2013 [13] Reference FDC	0.232	49.74 (20.14–92.12)	1
Current study, FDC tablets	---	19.94 (7.15–25.36)	0.330

AUC_0–24_: Area under the plasma concentration vs. time curve from 0 to 24 h obtained from the simulated plasma concentration using the models parameter estimates from the cited references as compared to the current study; AUC_0–24_ ratios: AUC_0–24_ by each model/AUC_0–24_ of the reference FDC by Milán-Segovia et al., 2013 [13]; Frel *: relative bioavailability estimates for the generic formulation used in the current study using priors from parameter estimates obtained previously in [13,14,15,17], which include a reference FDC tablet and different generic formulations. FDC: fixed-dose combination. Milán-Segovia et al., 2013 [13] reference FDC: Rifater, Sanofi-Aventis, Mexico City, Mexico (rifampicin + isoniazid + ethambutol + pyrazinamide). Milán-Segovia et al., 2013 [13] formulation A FDC: rifampicin + isoniazid + ethambutol + pyrazinamide FDC (unknown brand/manufacturer). Seng et al., 2015 [17]: RIF + Isoniazid FDC (unknown brand/manufacturer). Wilkins et al. 2008 [15]: RIF + isoniazid + ethambutol + pyrazinamide FDC (unknown brand/manufacturer). Current clinical trial and Schipani et al., 2016 [14]: RIF + isoniazid + ethambutol + pyrazinamide FDC (Lupin Pharmaceutical Ltd., Chikalthana, Aurangabad, India).

## Data Availability

Original data are available from the corresponding author upon request.

## Supplementary Materials

The following supporting information can be downloaded at https://www.mdpi.com/article/10.3390/pharmaceutics16080970/s1, **Figure S1**: Goodness of fit (GOF) plots of rifampicin (RIF) and 25-O-desacetyl-rifampicin (desRIF) by the final model. Observed concentrations (µg/mL) over population and individual predictions (right). Conditional weighted residuals (CWRESs) over population predictions and time (left). Red line: trend line, dashed lines in right plots: identity, and 2- and 0.5-times identity. Dashed lines in left plots: −2, 0 and 2 CWRESs. **Figure S2**: Posterior predictive check (PPC) of rifampicin pharmacokinetics model. Frequency histograms show the predicted distribution of the simulated AUC_0–24_ and C_max_ values (*n* = 1000 simulations). Red lines: 5th, 50th and 95th percentiles of the observed AUC_0–24_ and C_max_. Blue lines: 5th, 50th and 95th percentiles of the individual predicted AUC_0–24_ and C_max_. **Figure S2 (continuation)**: Posterior predictive check (PPC) of 25-O-desacetyl-rifampicin pharmacokinetics model. Frequency histograms show the predicted distribution of the simulated AUC_0–24_ and C_max_ values (*n* = 1000 simulations). The red lines depict the 5th, 50th and 95th percentiles of the observed AUC_0–24_ and C_max_; and blue lines depict the 5th, 50th and 95th percentiles of the individual predicted AUC_0–24_ and C_max_. **Figure S3**: Normalised predictive distribution errors (NPDE) of the model describing the pharmacokinetics of rifampicin and 25-O-desacetly-rifampicin. NPDE vs. predicted concentrations (top left) and time (top right). NPDE histogram (bottom left), NPDE normal quantile-quantile plot (bottom right). **Figure S4**: Mirror plots of rifampicin with the final model. Individual observed concentrations vs. population and individual predicted concentrations. Conditional weighed residuals (CWRESs) vs. population predicted concentrations and time. **Figure S4 (continuation)**: Mirror plots of 25-O-desacetly-rifampicin with the final model. Individual observed concentrations vs. population and individual predicted concentrations. Conditional weighed residuals (CWRESs) vs. population predicted concentrations and time. **Table S1**: Pharmacokinetic parameters of rifampicin (RIF) and 25-O-desacetyl-rifampicin (desRIF) using the model from Seng et al., 2015 [17] as priors. **Table S2**: Pharmacokinetic parameters of rifampicin (RIF) and 25-O-desacetyl-rifampicin (desRIF) using the model from Schipani et al., 2016 [14] as priors. **Table S3**: Pharmacokinetic parameters of rifampicin (RIF) and 25-O-desacetyl-rifampicin (desRIF) using the model from Wilkins et al., 2008 [15] as priors. **Table S4**: Pharmacokinetic parameters of rifampicin (RIF) and 25-O-desacetyl-rifampicin (desRIF) using the model from Milán-Segovia et al., 2013 [13] as priors.
