# Supplementary material for: Effect of Interindividual Variability in Metabolic Clearance and Relative Bioavailability on Rifampicin Exposure in Tuberculosis Patients with and without HIV Co-Infection: Does Formulation Quality Matter?
Source: Pharmaceutics. 2024 Jul 23;16(8):970. doi: 10.3390/pharmaceutics16080970 (PMC11359463; doi:10.3390/pharmaceutics16080970)
Supplement: Supplementary file 1 [file pharmaceutics-16-00970-s001.zip › Supplementary Material.pdf]

Effect of interindividual variability in metabolic clearance and relative bioavailability on rifampicin exposure in tuberculosis patients with and without HIV co-infection: does formulation quality matter?

SUPPLEMENTARY MATERIAL

Analytical method for quantification of rifampicin and 25-O-desacetyl-rifampicin in plasma

A liquid chromatography-tandem accurate mass spectrometry (LC-MS/MS) method was developed and validated to quantify rifampicin, 25-O-desacetyl-rifampicin in plasma with low limits of quantification. Full details of the method development and validation are described previously[1]. The coefficients of variation (CV) and relative standard errors (RSE) of intra-and inter-assay precision and accuracy were lower than 15%. Analytical linearity range of RIF and desRIF were 1.22–5000 and 1.95–1000 ng/ml (lower limit of quantification to upper boundary).

DNA extraction and genotyping of OATP1B1

Genomic DNA was extracted from whole blood using chaotropic salting out standard procedures. Taqman SNP genotyping assays and a Fast 7500 Real-Time System (Applied Biosystems, Foster City, CA, USA) were used for allele discrimination at the polymorphic loci of *SLCO1B1*: g.521T>C (rs4149056), g.463C>A (rs11045819), g.38664C>T (rs4149032)[2]. Allele and genotype frequency were derived by gene counting.

Model qualification

The goodness of fitting plots (GOF)[2,3] includes plots of the population predicted (PRED) and individual predicted (IPRED) concentrations versus the observed concentrations, and the conditional weighted residuals (CWRES) versus PRED and time (Figure S1).

Visual predictive check (VPC)[3–5], posterior predictive check (PPC)[6] and normalised predictive distribution errors (NPDE)[7] were obtained from 1000 simulations of plasma concentrations from 0 to 24 h for individual subjects with the same demographic characteristics, dosing regimens and sampling schedule as in the original clinical data. VPCs are shown in Figure 3 of the manuscript.

In addition, PPC was performed using the area under the plasma concentration vs time curve from 0-24h ($AUC_{0\text{-}24}$) and maximum plasma concentration (Cmax) as measures of model performance. Predicted and observed $AUC_{0\text{-}24}$ (trapezoidal method) and Cmax values were calculated non-compartmentally. The simulated $AUC_{0\text{-}24}$ and Cmax histograms are presented along with the median, 5th and 95th percentiles of observed $AUC_{0\text{-}24}$ and Cmax (Figure S2).

NPDEs were calculated using the 'npde' package v.2.0 in R in order to assess general model performance in subsequent simulations. The NPDE results were summarised graphically in (i) QQ-plot of the NPDE; (ii) histogram of the NPDE; (iii) NPDE versus time and (iv) NPDE versus predicted concentrations. The NPDE is expected to follow a N (0-1) distribution (Figure S3).

These results were complemented by mirror plots, which were generated in PsN. Mirror plots were aimed at assessing the degree of similarity across observed and simulated concentration vs. time profiles (Figure S4).

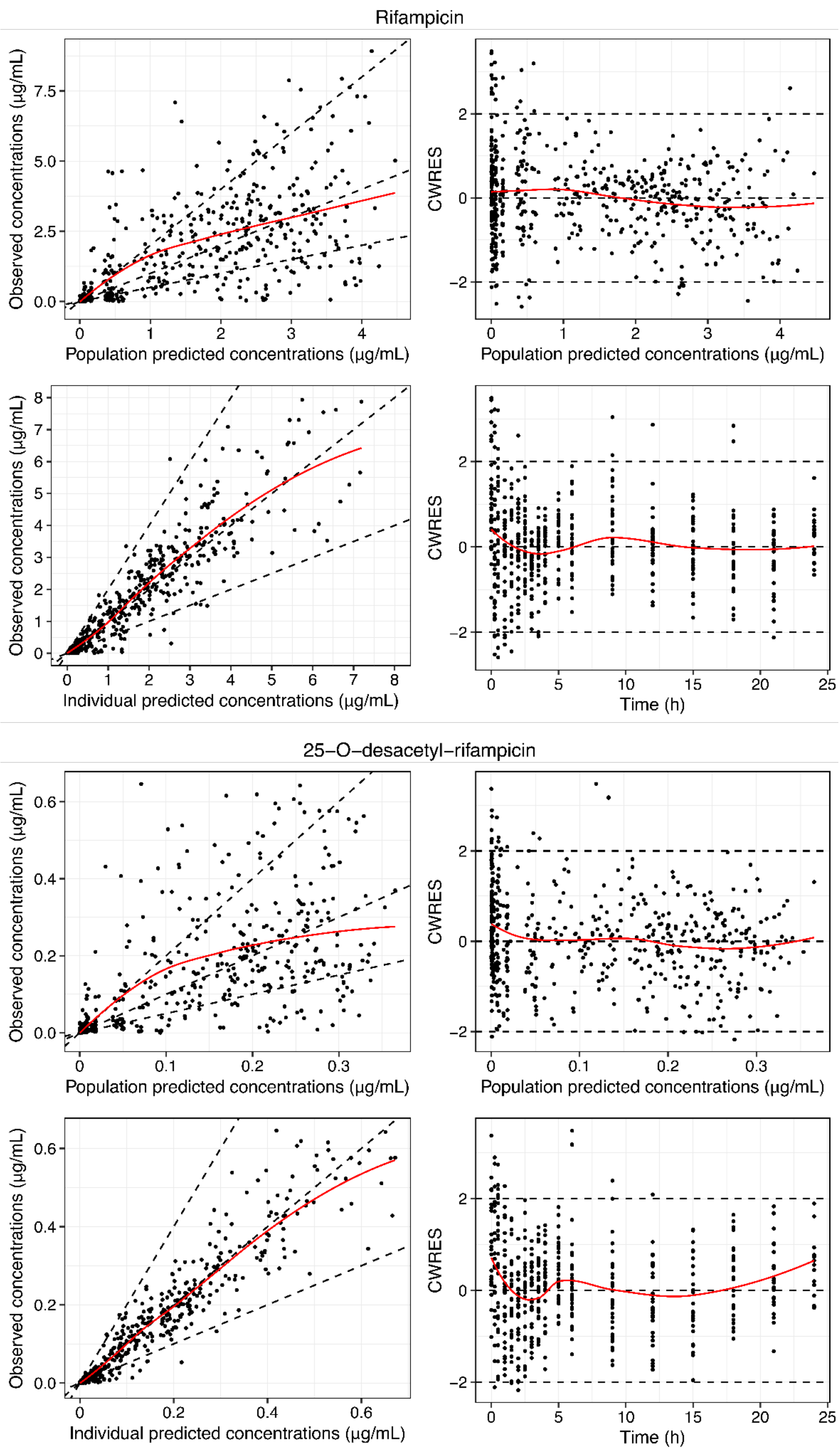

Figure S1: Goodness of fit plots (GOF) of rifampicin (RIF) and 25-O-desacetyl-rifampicin (desRIF) by the final model. Observed concentrations (µg/mL) over population and individual predictions (right). Conditional weighted residuals (CWRES) over population predictions and time (left). Red line: trend line, dashed lines in right plots: identity, and 2- and 0.5-times identity. Dashed lines in left plots: -2, 0 and 2 CWRES

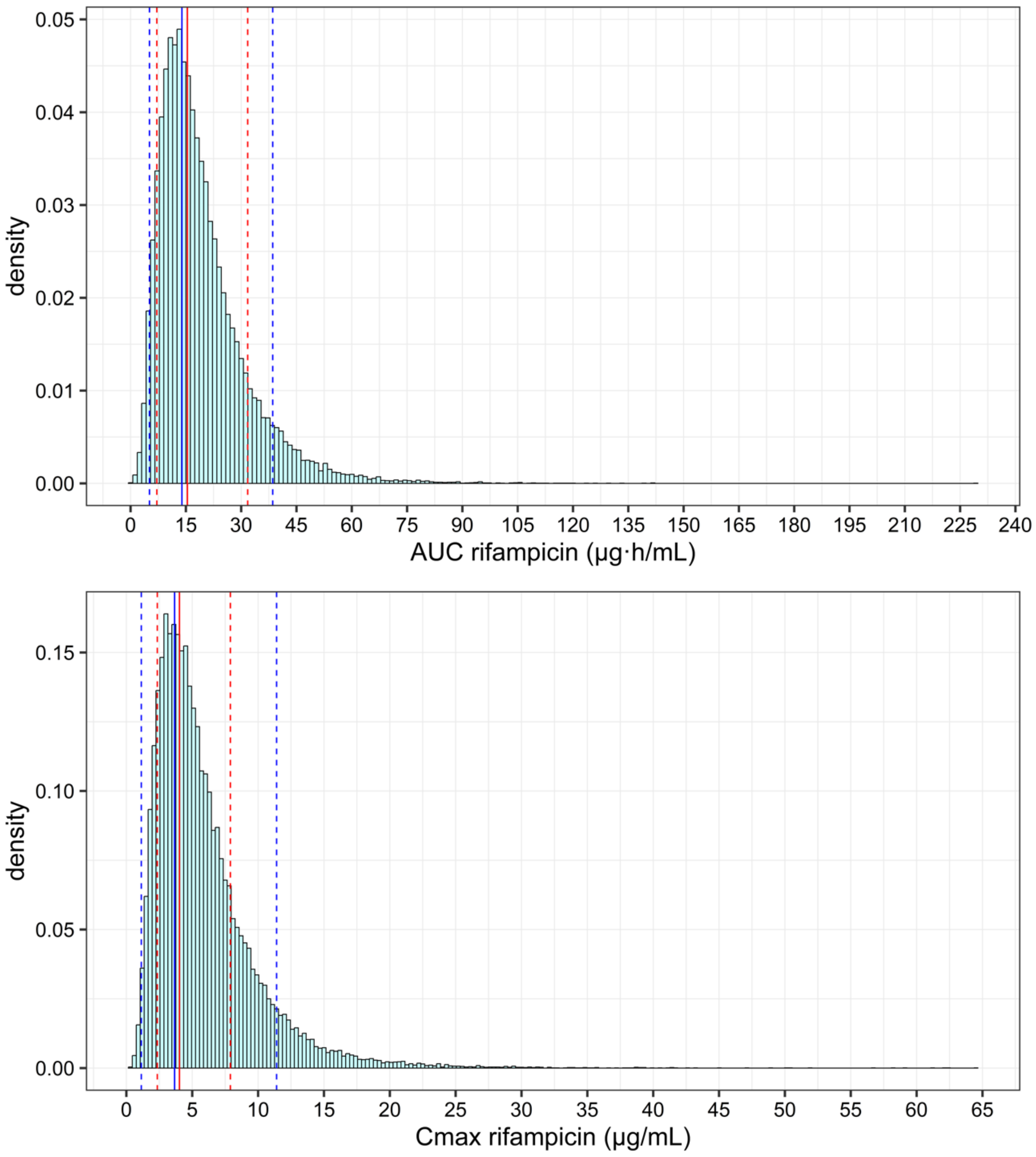

Figure S2: Posterior predictive check (PPC) of rifampicin pharmacokinetics model. Frequency histograms show the predicted distribution of simulated AUC$_{0-24}$ and Cmax values (n = 1000 simulations). Red lines: 5th, 50th and 95th percentiles of the observed AUC$_{0-24}$ and Cmax. Blue lines5th, 50th and 95th percentiles of the individual predicted AUC$_{0-24}$ and Cmax.

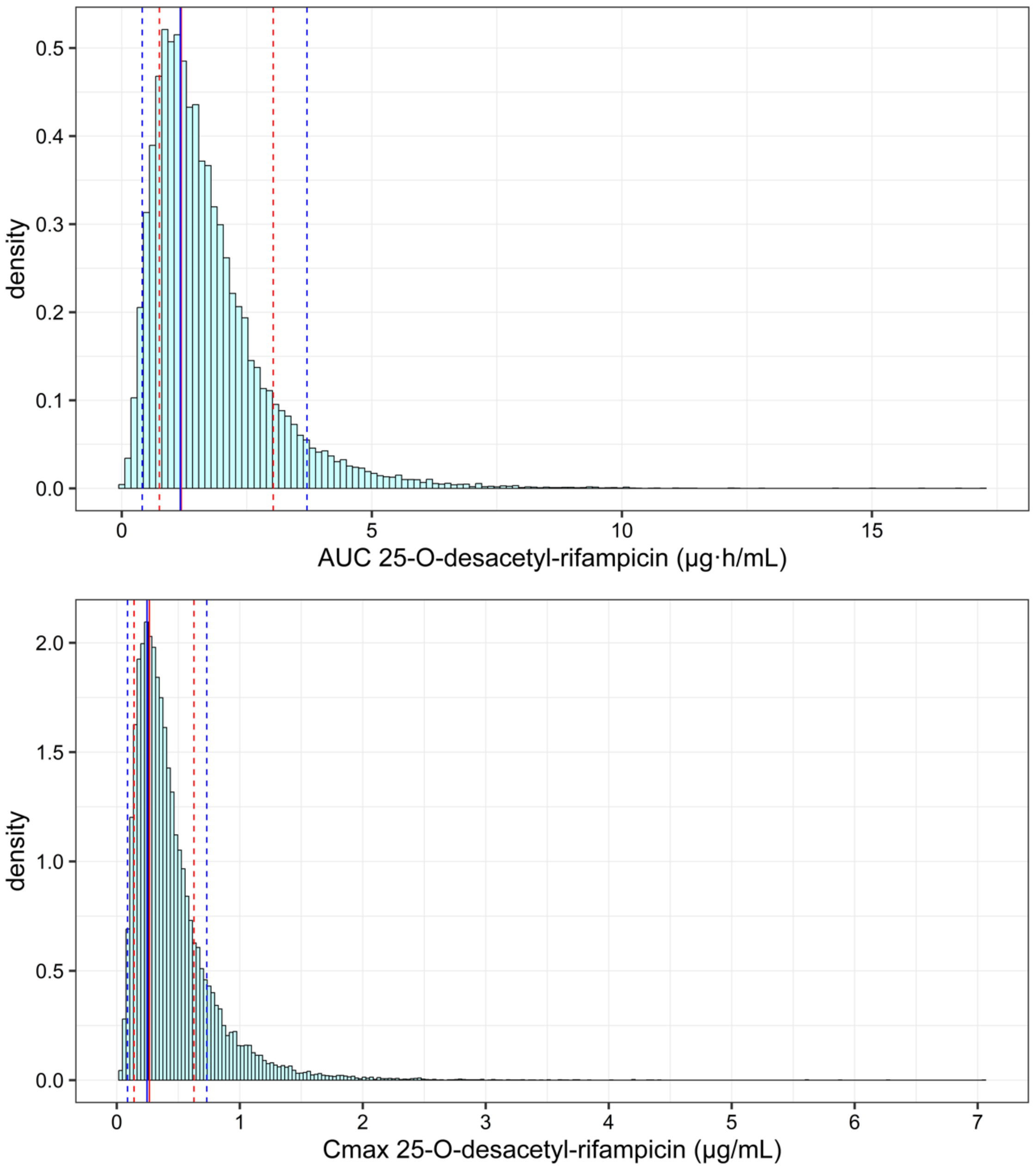

Figure S2 (continued): Posterior predictive check (PPC) of 25-O-desacetyl-rifampicin pharmacokinetics model. Frequency histograms show the predicted distribution of simulated AUC_{0-24} and Cmax values (n = 1000 simulations). The red lines depict the 5th, 50th and 95th percentiles of the observed AUC_{0-24} and Cmax; and blue lines depict the 5th, 50th and 95th percentiles of the individual predicted AUC_{0-24} and Cmax.

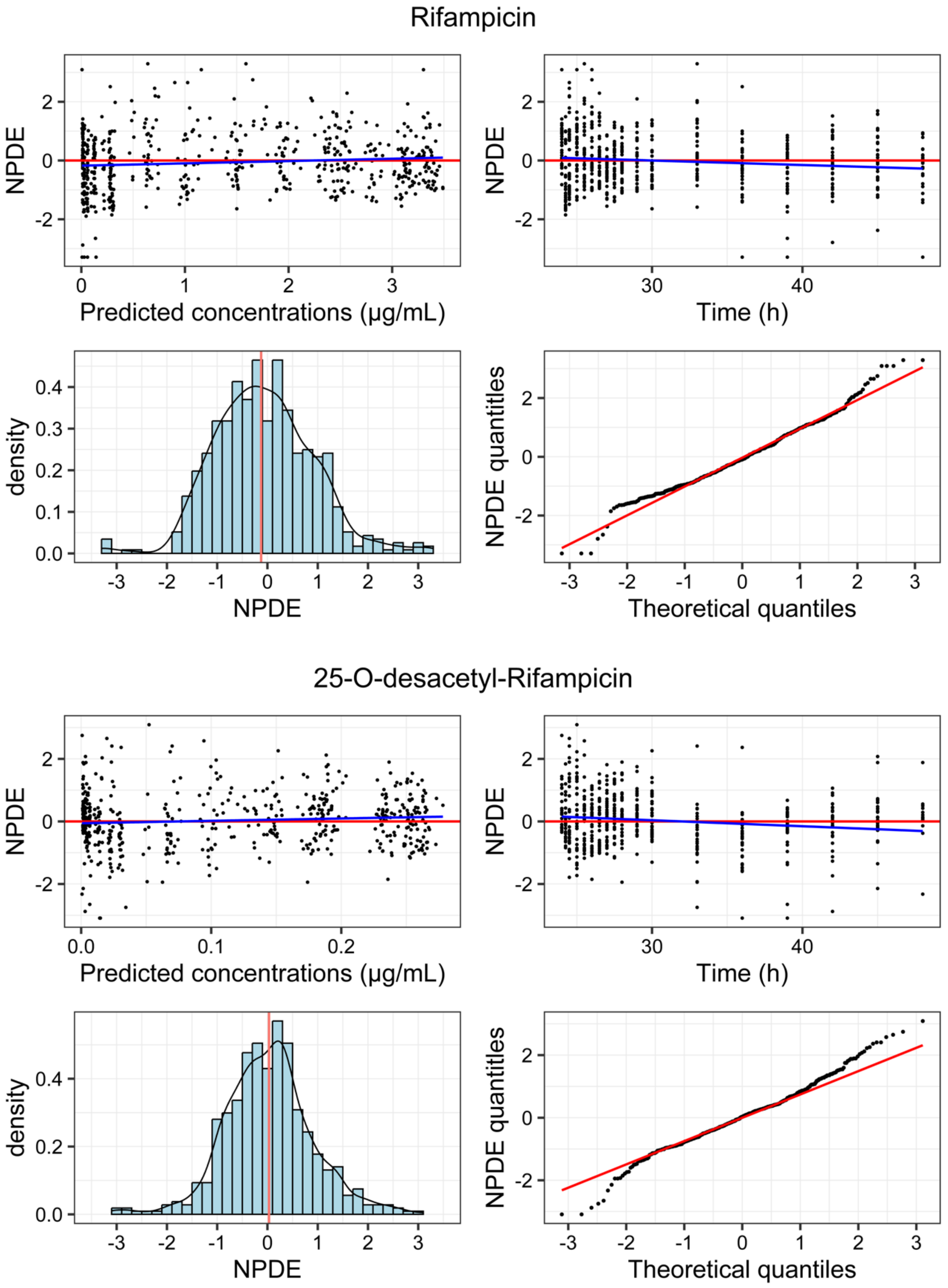

Figure S3: Normalised predictive distribution errors (NPDE) of rifampicin and 25-O-desacetly-rifampicin pharmacokinetics model. NPDE vs predicted concentrations (top left) and time (top right). NPDE histogram (bottom left) NPDE normal quantile – quantile plot (bottom right).

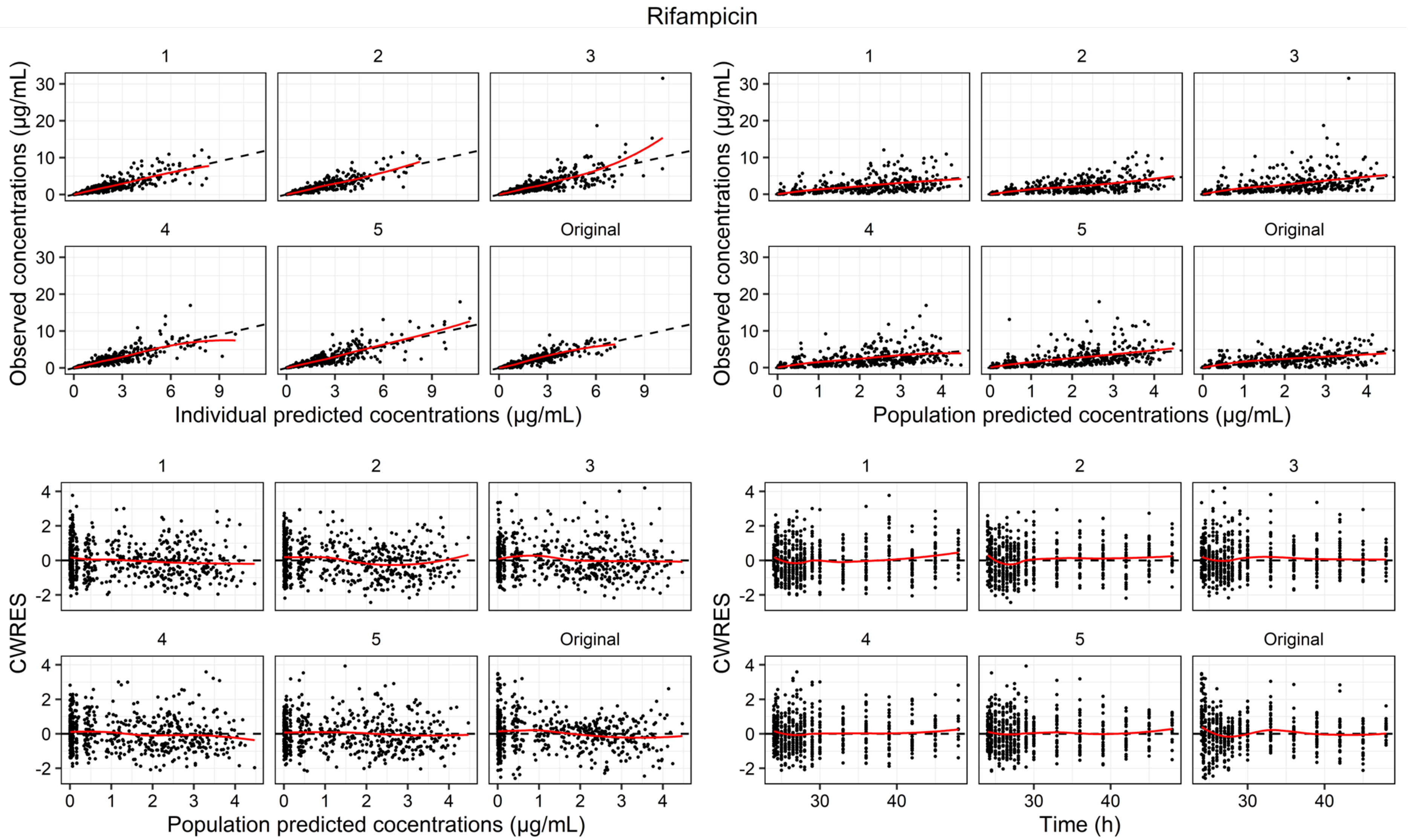

Figure S4: Mirror plots of rifampicin with the final model. Individual observed concentrations vs population and individual predicted concentrations. Conditional weighed residuals (CWRES) *vs* population predicted concentrations and time.

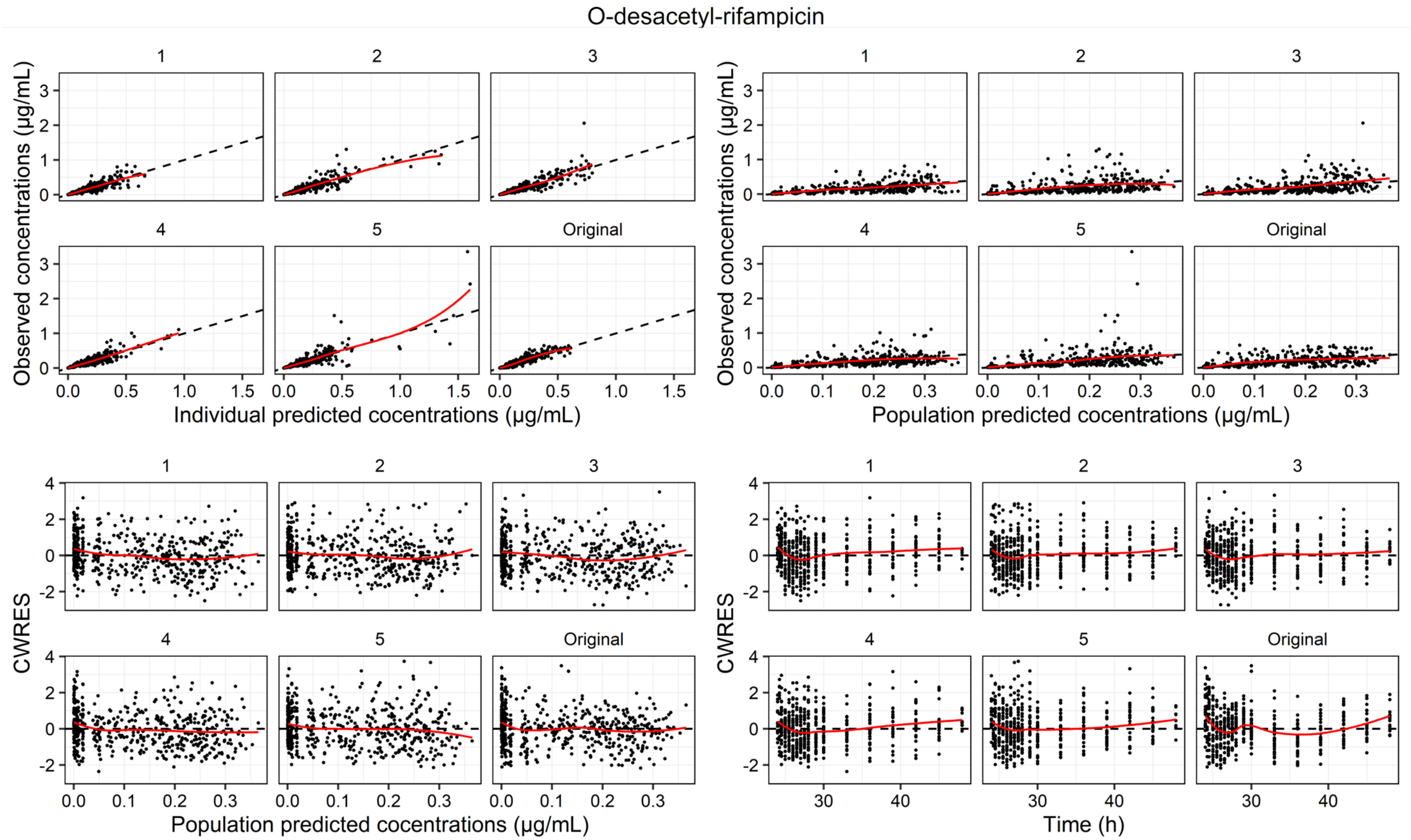

Figure S4 continued: Mirror plots of 25-O-desacetyl-rifampicin with the final model. Individual observed concentrations vs population and individual predicted concentrations. Conditional weighed residuals (CWRES) *vs* population predicted concentrations and time.

Table S1: Pharmacokinetic parameter estimates of rifampicin (RIF) and 25-O-desacetyl-rifampicin (desRIF) model including the model parameters from Seng et al., 2015[8] as priors.

Parameters	Typical value (RSE%)	IIV CV % (RSE%)
CL/F (L/h)	10.3 (0.3)	26.9 (10.6)
V/F (L)	31.5 (4.7)	
MTT (h)	1.13 (9.0)	53.2 (7.8)
Frel	0.292 (10.0)	46.9 (10.7)
nn	3 (fix)	
Fm	----	38.8 (15.8)
CLm/F·Fm (L/h)	108 (5.5)	
Vm/F·Fm (L)	66.1 (12.2)	63.3 (22.0)
Qm/F·Fm (L/h)	0 FIX	
Vpm/F·Fm (L/h)	0 FIX	
off-diagonal ω^2 CL/F - Fm		71.5 (2.3)
Residual error (ε)		
RIF proportional		45.6 (8.5)
RIF additive (nmol/mL)		1.85E-5 (38.6)
η_1*		20.4 (38.0)
desRIF proportional		35.5 (10.1)
desRIF additive (nmol/mL)		3.21E-6 (15.7)
η_2*		35.1 (28.4)

$\mathrm{CV\%} = \sqrt{\exp(\omega^2) - 1} \cdot 100$ or $= \sqrt{\exp(\varepsilon^2) - 1} \cdot 100$. * η_1 and η_2 are random deviations of individual i from the variance of ε, which is assumed to be the same for all subjects. RSE: relative standard error. IIV: interindividual variability

Model parameterisation:

Ka = Ktr = (nn+1)/MTT

$$\mathrm{CL/F} = \mathrm{CL/F}_{\mathrm{Tipical\ Value}} \cdot \left(\frac{\mathrm{WT}}{\mathrm{WT\ median}}\right)^{0.75} \cdot e^{\eta}$$

$$\mathrm{V/F} = \mathrm{V/F}_{\mathrm{Tipical\ Value}} \cdot \left(\frac{\mathrm{WT}}{\mathrm{WT\ median}}\right) \cdot e^{\eta}$$

$$\mathrm{Fm} = 1 \cdot e^{\eta}$$

$$\mathrm{CLm/F \cdot Fm} = \mathrm{CL/F \cdot Fm}_{\mathrm{Tipical\ Value}} \cdot \left(\frac{\mathrm{WT}}{\mathrm{WT\ median}}\right)^{0.75} \cdot e^{\eta}$$

$$\mathrm{Vm/F \cdot Fm} = \mathrm{V/F \cdot Fm}_{\mathrm{Tipical\ Value}} \cdot \left(\frac{\mathrm{WT}}{\mathrm{WT\ median}}\right) \cdot e^{\eta}$$

$$\mathrm{F} = \mathrm{Frel} \cdot e^{\eta}$$

CL/F and CLm/F·Fm: apparent clearance of RIF and desRIF, respectively. Fm: fraction of RIF that is converted into desRIF. Frel: relative bioavailability. Ka: RIF absorption rate constant. Ktr: RIF transit rate constant. MTT: mean transit time. nn: number of transit compartments. V/F and Vm/F·Fm: apparent volume of distribution of RIF and desRIF, respectively. WT: weight. η and ε random variables with mean 0 and variance ω^2 and σ^2.

Table S2: Pharmacokinetic parameter estimates of rifampicin (RIF) and 25-O-desacetyl-rifampicin (desRIF) model including the model parameters from Schipani et al., 2016[9] as priors.

Parameters	Typical value (RSE%)	IIV CV% (RSE%)
CL/F (L/h)	23.9 (0.3)	26.9 (10.6)
V/F (L)	73.1 (4.7)	
MTT (h)	1.13 (9.0)	53.2 (7.8)
Frel	0.678 (10.0)	46.9 (10.7)
nn	3 (fix)	
Fm	----	38.8 (15.8)
CLm/F·Fm (L/h)	250 (5.5)	
Vm/F·Fm (L)	153 (12.2)	63.3 (22.0)
off-diagonal ω^2 CL/F - Fm		71.5 (2.3)
Residual error (ε)		
RIF proportional		45.6 (8.5)
RIF additive (nmol/mL)		1.85E-5 (39.2)
η_1*		20.4 (38.4)
desRIF proportional		35.5 (10.1)
desRIF additive (nmol/mL)		3.21E-6 (15.7)
η_2*		35.1 (28.4)

$CV\% = \sqrt{\exp(\omega^2) - 1} \cdot 100$ or $= \sqrt{\exp(\varepsilon^2) - 1} \cdot 100$. * η_1 and η_2 are random deviations of individual i from the variance of ε, which is assumed to be the same for all subjects. RSE: relative standard error. IIV: interindividual variability.

Model parameterisation:

Ka = Ktr = (nn+1)/MTT

$$CL/F = CL/F_{\text{Tipical Value}} \cdot \left(\frac{WT}{WT\ median}\right)^{0.75} \cdot e^{\eta}$$

$$V/F = V/F_{\text{Tipical Value}} \cdot \left(\frac{WT}{WT\ median}\right) \cdot e^{\eta}$$

$$Fm = 1 \cdot e^{\eta}$$

$$CLm/F \cdot Fm = CL/F \cdot Fm_{\text{Tipical Value}} \cdot \left(\frac{WT}{WT\ median}\right)^{0.75} \cdot e^{\eta}$$

$$Vm/F \cdot Fm = V/F \cdot Fm_{\text{Tipical Value}} \cdot \left(\frac{WT}{WT\ median}\right) \cdot e^{\eta}$$

$$F = Frel \cdot e^{\eta}$$

CL/F and CLm/F·Fm: apparent clearance of RIF and desRIF, respectively. Fm: fraction of RIF that is converted into desRIF. Frel: relative bioavailability. Ka: RIF absorption rate constant. Ktr: RIF transit rate constant. MTT: mean transit time. nn: number of transit compartments. V/F and Vm/F·Fm: apparent volume of distribution of RIF and desRIF, respectively. WT: weight. η and ε random variables with mean 0 and variance ω^2 and σ^2.

Table S3: Pharmacokinetic parameter estimates of rifampicin (RIF) and 25-O-desacetyl-rifampicin (desRIF) model including the model parameters from Wilkins et al. 2008[10] as priors.

Parameters	Typical value (RSE%)	IIV CV& (RSE%)
CL/F (L/h)	19.2 (0.1)	28.2 (12.9)
V/F (L)	53.2 (0.1)	
MTT (h)	1.14 (9.0)	53.3 (7.9)
Frel	0.499 (9.3)	46.8 (10.6)
nn	3 (fix)	
Fm	----	40.2 (17.1)
CLm/F·Fm (L/h)	201 (5.8	
Vm/F·Fm (L)	124 (12.6)	63.4 (21.7)
off-diagonal ω^2 CL/F - Fm		73.8 (2.9)
Residual error (ε)		
RIF proportional		45.3 (8.5)
RIF additive (nmol/mL)		2.05E-5 (39.3)
η_1*		21.1 (36.8)
desRIF proportional		35.4 (10.0)
desRIF additive (nmol/mL)		3.28E-6 (15.4)
η_2*		35.4 (27.6)

$CV\% = \sqrt{\exp(\omega^2) - 1} \cdot 100$ or $= \sqrt{\exp(\varepsilon^2) - 1} \cdot 100$. * η_1 and η_2 are random deviations of individual i from the variance of ε, which is assumed to be the same for all subjects. RSE: relative standard error. IIV: interindividual variability.

Model parameterisation:

Ka = Ktr = (nn+1)/MTT

$$CL/F = CL/F_{\text{Tipical Value}} \cdot \left(\frac{WT}{WT\ \text{median}}\right)^{0.75} \cdot e^{\eta}$$

$$V/F = V/F_{\text{Tipical Value}} \cdot \left(\frac{WT}{WT\ \text{median}}\right) \cdot e^{\eta}$$

$$Fm = 1 \cdot e^{\eta}$$

$$CLm/F \cdot Fm = CL/F \cdot Fm_{\text{Tipical Value}} \cdot \left(\frac{WT}{WT\ \text{median}}\right)^{0.75} \cdot e^{\eta}$$

$$Vm/F \cdot Fm = V/F \cdot Fm_{\text{Tipical Value}} \cdot \left(\frac{WT}{WT\ \text{median}}\right) \cdot e^{\eta}$$

$$F = Frel \cdot e^{\eta}$$

CL/F and CLm/F·Fm: apparent clearance of RIF and desRIF, respectively. Fm: fraction of RIF that is converted into desRIF. Frel: relative bioavailability. Ka: RIF absorption rate constant. Ktr: RIF transit rate constant. MTT: mean transit time. nn: number of transit compartments. V/F and Vm/F·Fm: apparent volume of distribution of RIF and desRIF, respectively. WT: weight. η and ε random variables with mean 0 and variance ω^2 and σ^2.

Table S4: Pharmacokinetic parameter estimates of rifampicin (RIF) and 25-O-desacetyl-rifampicin (desRIF) model including the model parameters from Milán-Segovia et al., 2013[11] as priors.

Parameters	Typical value (RSE%)	IIV CV% (RSE%)
CL/F (L/h)	8.17 (0.1)	26.9 (10.6)
V/F (L)	25 (4.7)	
MTT (h)	1.13 (9.0)	53.2 (7.8)
Frel formulation A	0.497 (10)	47.1 (10.6)
Frel reference*	0.232 (10)	46.9 (10.7)
nn	3 (fix)	
Fm	----	38.8 (15.8)
CLm/F·Fm (L/h)	85.3 (5.5)	
Vm/F·Fm (L)	52.3 (12.2)	63.3 (21.9)
off-diagonal ω^2 CL/F - Fm		71.5 (2.3)
Residual error (ε)		
RIF proportional		45.6 (8.4)
RIF additive (nmol/mL)		1.84E-5 (38.9)
η_1*		20.4 (37.7)
desRIF proportional		35.5 (10.0)
desRIF additive (nmol/mL)		3.20E-6 (15.8)
η_2*		35.1 (28.3)

$\mathrm{CV\%} = \sqrt{\exp(\omega^2) - 1} \cdot 100$ or $= \sqrt{\exp(\varepsilon^2) - 1} \cdot 100$. * η_1 and η_2 are random deviations of individual i from the variance of ε, which is assumed to be the same for all subjects. RSE: relative standard error. IIV: interindividual variability.

Model parameterisaion:

Ka = Ktr = (nn+1)/MTT

$$\mathrm{CL/F} = \mathrm{CL/F}_{\mathrm{Tipical\ Value}} \cdot \left(\frac{\mathrm{WT}}{\mathrm{WT\ median}}\right)^{0.75} \cdot e^{\eta}$$

$$\mathrm{V/F} = \mathrm{V/F}_{\mathrm{Tipical\ Value}} \cdot \left(\frac{\mathrm{WT}}{\mathrm{WT\ median}}\right) \cdot e^{\eta}$$

$$\mathrm{Fm} = 1 \cdot e^{\eta}$$

$$\mathrm{CLm/F \cdot Fm} = \mathrm{CL/F \cdot Fm}_{\mathrm{Tipical\ Value}} \cdot \left(\frac{\mathrm{WT}}{\mathrm{WT\ median}}\right)^{0.75} \cdot e^{\eta}$$

$$\mathrm{Vm/F \cdot Fm} = \mathrm{V/F \cdot Fm}_{\mathrm{Tipical\ Value}} \cdot \left(\frac{\mathrm{WT}}{\mathrm{WT\ median}}\right) \cdot e^{\eta}$$

$$\mathrm{F} = \mathrm{Frel} \cdot e^{\eta}$$

CL/F and CLm/F·Fm: apparent clearance of RIF and desRIF, respectively. Fm: fraction of RIF that is converted into desRIF. Frel: relative bioavailability. Ka: RIF absorption rate constant. Ktr: RIF transit rate constant. MTT: mean transit time. nn: number of transit compartments. V/F and Vm/F·Fm: apparent volume of distribution of RIF and desRIF, respectively. WT: weight. η and ε random variables with mean 0 and variance ω^2 and σ^2.

References

1. Nardotto GHB, Bollela VR, Rocha A, Della Pasqua O, Lanchote VL. No implication of HIV coinfection on the plasma exposure to rifampicin, pyrazinamide, and ethambutol in tuberculosis patients. *Clin Translat Sci*. 2022; 15(2):514-523.

2. Weiner M, Peloquin C, Burman W; Luo CC; Engle M, Prihoda TJ, Kenzie WRM, Bliven-Sizemore E, Johnson JL, Vernon A. Effects of tuberculosis, race, and human gene SLCO1B1 polymorphisms on rifampin concentrations. *Antimicrob. Agents Chemother.* 2010, 54:4192–4200.

3. Mould DR, Upton RN. Basic concepts in population modeling, simulation, and model-based drug development—Part 2: introduction to pharmacokinetic modeling methods. *CPT Pharmacometrics Syst Pharmacol*. 2013;2(4): e38.

4. Nguyen THT, Mouksassi MS, Holford N, et al. Model evaluation of continuous data Pharmacometric models: metrics and graphics. *CPT: Pharmacometrics Syst Pharmacol*. 2017; 6(2):87-109.

5. Bergstrand M, Hooker AC, Wallin JE, Karlsson MO. Prediction-corrected visual predictive checks for diagnosing nonlinear mixed-effects models. *AAPS J*. 2011; 13(2):143-151.

6. Yano Y, Beal SL, Sheiner LB. Evaluating pharmacokinetic/pharmacodynamic models using the posterior predictive check. *J Pharmacokinet Pharmacodyn*. 2001; 28(2):171-192.

7. Comets E, Brendel K, Mentré F. Computing normalised prediction distribution errors to evaluate nonlinear mixed-effect models: The npde add-on package for R. *Comp Method Program Biomed*. 2008; 90(2):154-166.

8. Seng KY, Hee KH, Soon GH, Chew N, Khoo SH, Lee LSU. Population pharmacokinetics of rifampicin and 25-desacetyl-rifampicin in healthy Asian adults. *J Antimicrob Chemother*. 2015; 70(12):3298-3306.

9. Schipani A, Pertinez H, Mlota R, et al. A simultaneous population pharmacokinetic analysis of rifampicin in Malawian adults and children. *Br J Clin Pharmacol*. 2016;81(4):679-687. doi:10.1111/bcp.12848

10. Wilkins JJ, Savic RM, Karlsson MO, et al. Population Pharmacokinetics of rifampin in pulmonary tuberculosis patients, including a semimechanistic model to describe variable absorption. *Antimicrob Agents Chemother*. 2008; 52(6):2138-2148.

11. Milán-Segovia RC, Ramírez AMD, Cook HJ, et al. Population pharmacokinetics of rifampicin in Mexican patients with tuberculosis. *J Clin Pharm Ther*. 2013; 38(1):56-61.

NONMEM control file of rifampicin and O-desacetyl-rifampicin model

```
$PROBLEM
$INPUT     ID TIME ; hour
           AMT ; mmol
           SS II DV ; nmol/mL
           MDV CMT WEIGHT ; Kg
           HEIGHT ; metres
           AGE ; years
           HIV SEX
$DATA     RIF&DESRIF_21_07_13_l2_mol.csv IGNORE=@
$SUBROUTINE ADVAN6 TOL=6
$MODEL     NCOMP=6
$PK
;; free fat mass calculation ;;;;
IF (SEX.EQ.0) FFM=(42.92*(HEIGHT**2)*WEIGHT)/((HEIGHT**2)*30.93+WEIGHT)
IF (SEX.EQ.1) FFM=(37.99*(HEIGHT**2)*WEIGHT)/((HEIGHT**2)*35.98+WEIGHT)

F1=1*EXP(ETA(4))                                  ;; RIF bioavailability
CL=THETA(1)*((WEIGHT/55.7)**0.75)*EXP(ETA(1))     ;; RIF clearance
V=THETA(2)*(WEIGHT/55.7)*EXP(ETA(6))              ;; RIF volume of distribution
FM=THETA(3)*EXP(ETA(2))                      ;; fraction converted to desRIF
CLM=THETA(4)*((WEIGHT/55.7)**0.75)*EXP(ETA(5))    ;; desRIF clearance
VM=THETA(6)*(WEIGHT/55.7)*EXP(ETA(7))             ;; desRIF volume of distribution

S2=V/1000
S3=VM/1000

 ;;RIF absorption transit model
MTT=THETA(5)*EXP(ETA(3))
NN=3
KTR=((NN+1)/MTT)
KA=KTR

$DES
DADT(1)=-KA*A(1)
DADT(2)=KTR*A(6)-(CL*(1-FM)/V)*A(2)-(CL*FM/V)*A(2)
DADT(3)=(CL*FM/V)*A(2)-(CLM/VM)*A(3)
DADT(4)=KA*A(1)-KTR*A(4)
DADT(5)=KTR*A(4)-KTR*A(5)
DADT(6)=KTR*A(5)-KTR*A(6)

$ERROR
IF(CMT.EQ.2) THEN
IPRED=A(2)/S2
EPSrif=IPRED*EPS(1)*EXP(ETA(9))
Y=IPRED+EPSrif+EPS(3)
END IF

IF(CMT.EQ.3) THEN
IPRED=A(3)/S3
EPSdesrif=IPRED*EPS(2)*EXP(ETA(8))
```

```
Y=IPRED+EPSdesrif+EPS(4)
END IF

IF (CMT.EQ.2) STRT=2
IF (CMT.EQ.3) STRT=3

$THETA  (0,35.2446)      ; CL
 (0,107.724)             ; V
 1 FIX                   ; FM
 (0,368.409)             ; CLM
 (0,1.13401)             ; MTT
 (0,226.079)             ; VM
$OMEGA  BLOCK(2)
 0.0701452                 ;  CL_
 -0.0707649 0.139866     ; FM_

$OMEGA  0.248705          ; MTT_
 0.199309                  ; F
 0  FIX                    ; CLm
 0  FIX                    ; V_
 0.337316                  ; Vm
 0.116391                  ; RUV-desrif
 0.0408906                 ; RUV-rif

$SIGMA  0.188939          ; prop
 0.118984                  ; prop-desrif
$SIGMA  1.84976E-005    ; add
 3.20746E-006              ; add-desrif
$ESTIMATION METHOD=1 INTER MAXEVAL=99999 SIGL=6 NSIG=2 PRINT=1 NOABORT
$COVARIANCE UNCONDITIONAL
;$SIMULATION (20030521) ONLYSIM SUBPROBLEMS=1000
$TABLE
```
